# 2015 – a seminal year for HIV biomedical prevention

**DOI:** 10.7448/IAS.18.1.20814

**Published:** 2015-12-01

**Authors:** Iryna B Zablotska, Tim Spelman, Andrew Grulich

**Affiliations:** 1 The Kirby Institute, The University of New South Wales, Sydney, NSW, Australia; 2 Centre for Population Health, Burnet Institute, Melbourne, VIC, Australia

The past 5 years has been a time of increasing optimism in HIV treatment and HIV prevention. In 2015, two of the key chinks in the armour of biomedical prevention of HIV have been filled, with new evidence on the personal health benefit of early HIV treatment, and on the real-world efficacy of pre-exposure prophylaxis.

Prior to 2015, early initiation of antiretroviral treatment (ART) was recommended for prevention of onwards transmission of HIV, but randomized trial evidence supporting a health benefit of early treatment was lacking [1]. Earlier this year, the Strategic Timing of AntiRetroviral Treatment (START) trial reported evidence that immediate treatment of HIV upon diagnoses was associated with a 57% (confidence interval: 38 to 70%) reduction in any serious AIDS-related event, serious non-AIDS-related event or death from any cause [2]. This large reduction in adverse health consequences provides a compelling reason for recommending immediate HIV treatment at diagnosis for all people with HIV, regardless of CD4 count. Data on both the personal health and transmission–prevention effect of early HIV therapy are now in complete agreement: immediate treatment should be offered to all people with HIV regardless of the stage of infection. This new data on personal health benefit has led to significant changes in antiretroviral (ARV) guidelines, and for the first time developed country guidelines in Europe [3], the United States [4] and Australia [5] are in accord that all people with HIV should be offered immediate treatment.

In the field of pre-exposure prophylaxis (PrEP), the last 6 years have seen an escalation of evidence, including clinical trials and observational studies confirming the safety and efficacy of daily tenofovir disoproxil fumarate (TDF), alone or in combination with emtricitabine (TDF + FTC, marketed by Gilead Sciences as Truvada^®^) as PrEP [6]. PrEP has been shown to be effective in different population groups and across different settings. In 2015, two landmark studies, IPERGAY in France and PROUD in the UK, reported that PrEP reduced HIV risk among high-risk homosexual men by 86% [7,8]. This level of PrEP efficacy was much higher than it had been anticipated [8]. Both studies attracted extremely high-risk gay men, with annual incidence among the groups who did not receive PrEP of 8.9 and 6.6% in the UK and France, respectively. The high level of efficacy alleviated concerns that high levels of PrEP adherence may not be achievable [9]. It has become evident that those most attracted and motivated to use PrEP are people at highest risk for HIV, and motivated users are most likely to use PrEP appropriately [10,11]. PrEP for high-risk populations is now recommended in national or regional guidelines in the United States, Europe and Australia [3,12–14].

In 2015, the idea of using ART for both treatment and prevention of HIV has started moving from theory to implementation, but thus far only in a few locations. Encouraging examples in the United States include San Francisco, where the roll-out of early ART and PrEP, coupled with the high levels of testing for HIV, has coincided with a downturn of new HIV diagnoses [15]; the investment of the state government in a Pre-Exposure Prophylaxis Assistance Program (PrEP-AP) in New York, USA [16]; the rapid increases in PrEP use and its high effectiveness emerging from the health insurance data [17]. Outside of the United States, wide implementation of PrEP has been limited by the fact that Truvada is not registered for prevention, but applications have been lodged in Australia, Brazil, South Africa and Thailand and approvals are expected in 2016.

Also among this year's achievements is the rapid development of policy on biomedical prevention. Prior to 2015 guidance on PrEP was issued only by the United States, South Africa and World Health Organization (WHO) [12,18,19]. This year, new national PrEP guidelines have been released by the Australian Society for HIV Medicine [13] and the European AIDS Clinical Society (EACS [3]). Interestingly, the EACS guidelines for the first time recommended not just daily but also “on demand PrEP” (double dose of drug 2 to 24 hours before each sexual intercourse, followed by two single doses of drug, 24 and 48 hours after the first drug intake based on the regimen investigated by the European IPERGAY study [7]). Also anticipated by the end of 2015 is the release of the new, updated, consolidated ARV guidelines from the WHO which will expand on the broader implementation of PrEP [20]. Finally, the successes in biomedical prevention have re-energized community activism, with growing advocacy for broader access to ARVs, particularly as PrEP [21].

HIV testing is the gateway to both HIV treatment and prevention. HIV testing, early treatment and PrEP work well in synergy and each boosts implementation of the others. HIV testing, a triage to treatment or prophylaxis is itself expected to become more frequent as regular follow-up of ART users is recommended (including PrEP users) [12,20]. Furthermore, the biomedical prevention trio provides new opportunities to engage in prevention and care those people who are at the highest risk of acquiring or transmitting HIV [22]. Meanwhile, global coverage with ART of all people living with HIV has surpassed 40% [23], and UNAIDS has identified HIV testing as major goal to achieve better population coverage with ART and prevention worldwide [24]. Further improvements along the continuum of HIV services can be expected.

The new HIV prevention environment may be associated with some downsides. A concern has been raised that effective HIV prevention may be accompanied by decreases in the use of condoms among the high-risk population groups and a rise in STI diagnoses. So far, little clinical trial evidence suggests this is the case [25,26]. However, these are important concerns to consider. It is only logical to anticipate that condom use will decline with the fading threat of the global HIV epidemic. Over the last 10 to 15 years, as HIV treatment efficacy has increased, declines in condom use have been observed in some communities of homosexual men worldwide [27]. However, these declines in condom use, if offset by the adequate levels of coverage with biomedical prevention methods, may have no impact on HIV diagnoses. One of the key limitations on the real-world effectiveness of biomedical prevention is how rapidly we can increase the population-level implementation of improved HIV testing, immediate ART in people diagnosed with HIV and PrEP for high-risk populations. True population-level access to the novel testing and treatment options remains a challenge in many settings, particularly access to PrEP outside the United States. At the same time, mass availability of tests and medications are not the only pre-requisites necessary for rapid roll-out of the recent biomedical advances. Implementation programmes need to target marginalized and criminalized populations, to ensure that these groups have opportunities to benefit from access to HIV testing and linkage to care. These are settings, where policy and legal reforms are still necessary to remove existing punitive laws, engage civil society in implementation processes and programmes, and develop programmes and meaningful collaborations with key populations to hear their voices. A good example of work in this area is the regional consultation of the Asia Pacific Coalition on Male Sexual Health (APCOM), focused on leadership, advocacy and community involvement in preparing for the roll-out of PrEP [28].

This year's International AIDS Day marks a time when biomedical approaches, in combination with long-standing behavioural approaches, are assuming a key role in HIV prevention. The focus is now shifting to implementation research. This is the time for developing new PrEP options and their delivery mechanisms for primary HIV prophylaxis; the time to focus on developing an effective HIV vaccine and a cure and to focus on old and resurging issues such as the epidemics of sexually transmitted infections.
